# Delayed spontaneous resolution of a double anterior chamber following deep anterior lamellar keratoplasty (DALK)

**DOI:** 10.1186/s12886-024-03819-6

**Published:** 2024-12-30

**Authors:** Poramaporn Luangprasert, Passara Jongkhajornpong, Kaevalin Lekhanont, Manachai Nonpassopon, Varintorn Chuckpaiwong

**Affiliations:** 1 Department of Ophthalmology, Faculty of Medicine Ramathibodi Hospital, Mahidol University, Bangkok, Thailand; 2 Department of Ophthalmology, Somdetphraphutthaloetla Hospital, Samut Songkhram, Thailand

**Keywords:** Double anterior chambers, Descemet membrane detachment, Deep anterior lamellar keratoplasty, Descemetopexy

## Abstract

**Background:**

This study reports a rare case of delayed spontaneous resolution of double anterior chambers (AC) resulting from non-rhegmatogenous Descemet membrane detachment (DMD) after deep anterior lamellar keratoplasty (DALK). Currently, management guidelines for this condition have not been established.

**Case presentation:**

A 65-year-old woman with lattice corneal dystrophy underwent uncomplicated DALK, during which an unrecognized type 2 big bubble was present. On postoperative day 1, a total DMD was observed, and descemetopexy was attempted. After an unsuccessful air-filled descemetopexy, we opted for observation without further intervention. Spontaneous reattachment of Descemet membrane with a clear cornea was achieved at 3 months postoperatively. The endothelial cell count was 2,165 cells/ mm^2^.

**Conclusions:**

The spontaneous resolution of double AC in patients with DMD without visible perforation after DALK suggests that a “wait and observe” approach can be a reasonable and effective management strategy.

## Background

Deep anterior lamellar keratoplasty (DALK) has become the preferred surgical option for improving vision in corneal stromal disorders that spare the Descemet membrane (DM) and endothelium, such as ectatic disorders, corneal opacities, dystrophies and degenerations [1]. This selective replacement of the pathological anterior cornea while preserving the normal endothelium offers significant advantages over penetrating keratoplasty (PKP), particularly by eliminating endothelial rejection and prolonging graft survival [2]. Among several techniques, the big-bubble (BB) technique remains the most reliable approach, achieving the deepest, smoothest, and most uniform recipient surface, providing visual outcomes comparable to PKP [3]. Other lamellar dissection techniques are valuable in cases where BB formation failed or is contraindicated [4]. 

Despite its advantages, DALK presents technical challenges and unique complications. A double anterior chamber (AC) formation is the most common early postoperative complication [5], typically associated with intraoperative perforation [6]. In most cases, descemetopexy is recommended to drain the interface fluid and seal the perforation, with a high success rate for reattachment [5]. However, this procedure can result in a mean endothelial cell loss of over 20% [7], and in unsuccessful cases, a second keratoplasty may be required. Management guidelines in Descemet membrane detachment (DMD) without visible perforation after DALK not have been established. We present a rare case of delayed spontaneous resolution of a double anterior chamber following DALK.

## Case presentaton

A 65-year-old Thai female was diagnosed with lattice corneal dystrophy. The patient underwent DALK in her right eye (RE) 15 years ago, and the corneal graft remained clear. Her best-corrected visual acuity (BCVA) was 20/70 in the RE and 20/200 in the left eye (LE). Anterior segment optical coherence tomography of the LE revealed that the corneal lesion extended from the anterior to the posterior stroma, without involvement of the DM-endothelial complex. Non-contact specular microscopy showed endothelial cell density of 1,180 cells/ mm^2^ and a central corneal thickness of 564 microns in the RE. The LE could not be measured due to dense stromal deposits. Based on clinical and instrumental findings, we decided to perform DALK on the LE. During surgery, the BB technique was attempted three times without success, leading to a conversion to manual dissection. The corneal stroma was filled with tiny air bubbles, becoming opaque and white. Dissection was carefully refined using a blunt-tipped iris, a 15-degree blade, and corneal stromal scissors, gradually removing layers until only a few air bubbles remained and a clear, uniform stromal surface was achieved. Manual DALK was successfully completed without rupture of the DM.

**Fig. 1:**
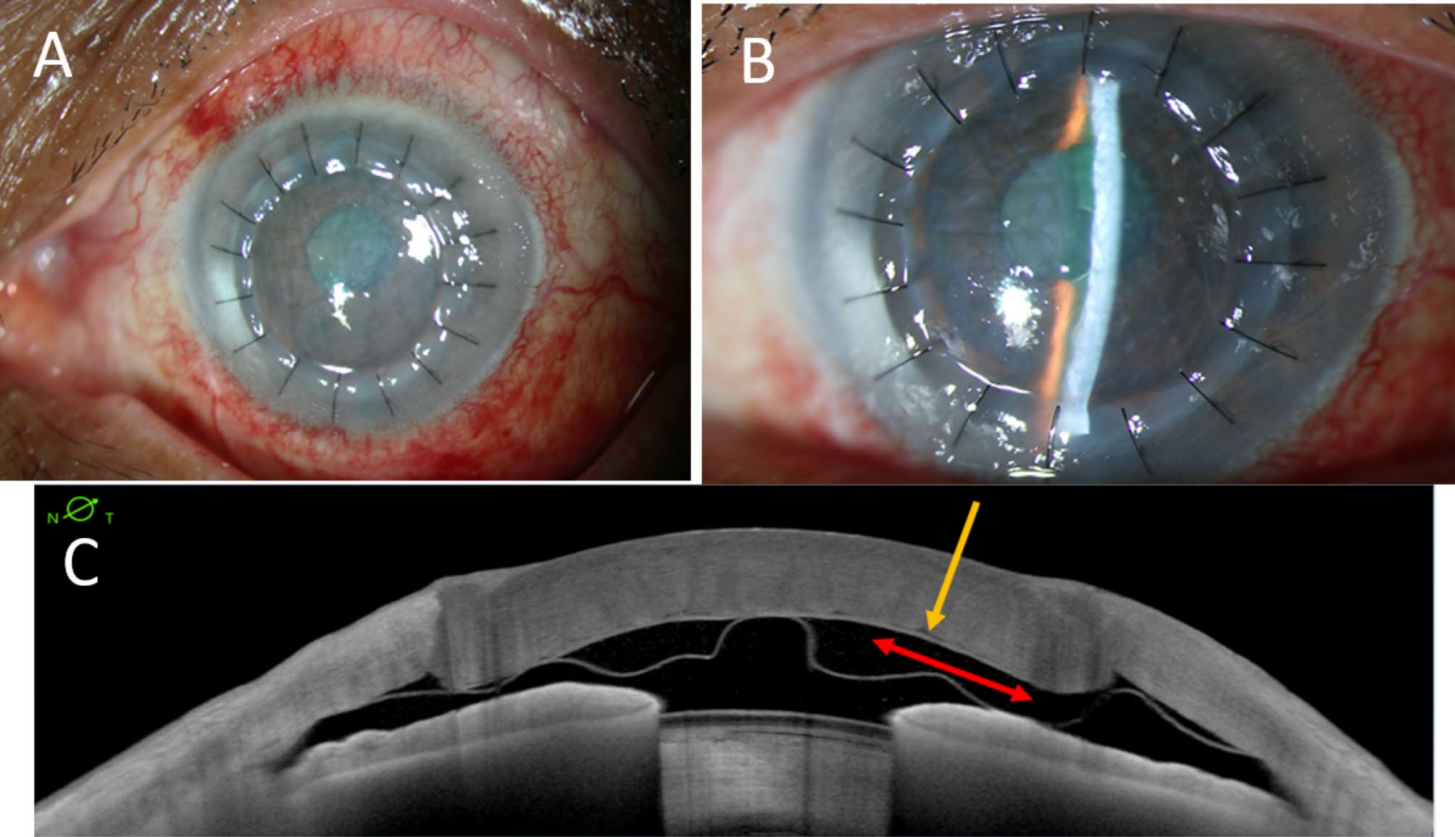
Anterior segment photos showed edematous cornea (**A**) due to Descemet membrane-endothelial complex detachment forming a double anterior chamber (**B**). Anterior segment optical coherence tomography (**C**) illustrates the Descemet membrane-endothelium complex detachment without a tear

**Fig. 2:**
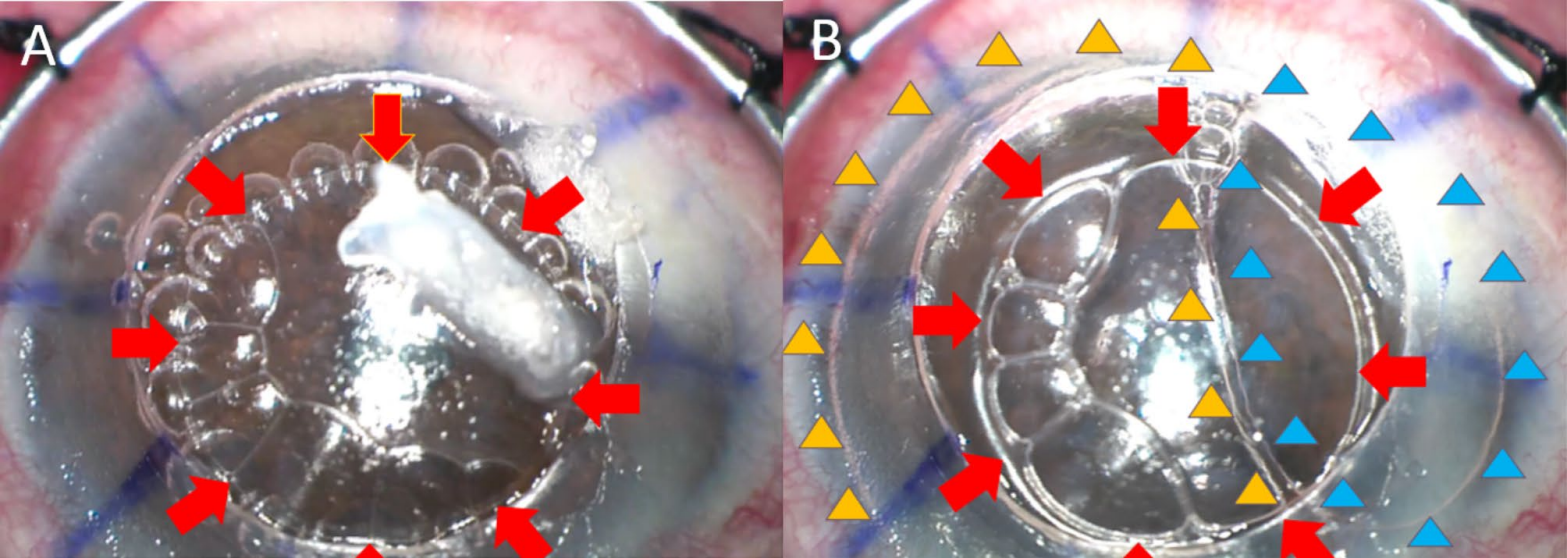
The type 2 bubble (red arrows) is surrounded by multiple small intracameral bubbles (**A**). The same type 2 big bubble was located above two large intracameral bubbles (yellow and blue arrowheads) that were injected subsequently (**B**).

On post-operative day 1, the DM-endothelial complex was found to be completely detached from the posterior stroma, resulting in a double anterior AC (Fig. 1A and B). The patient’s visual acuity was 20/160, and intraocular pressure was 6 mmHg. AS-OCT confirmed the diagnosis of DMD without DM tear (Fig. 1C). Upon reviewing the surgical video, an unrecognized BB type 2 was observed during the second attempt of air injection, which enlarged after the third attempt of air injection (Fig. 2A and B). The patient subsequently underwent an air injection with inferior peripheral iridotomy (PI), however, complete air fill of the AC could not be achieved due to the development of intraoperative malignant glaucoma. Malignant glaucoma occurred during the formation of the anterior chamber with balanced salt solution. We suspected that unintentinal placement of the needle too deep through the inferior PI leading to misdirection of aqueous humor into posterior space. Despite a patent 1-mm inferior PI at the 6 o’clock position, the anterior chamber shallowed, and the globe became tense. Air injection could not be performed. The elevated IOP was controlled with oral acetazolamide and topical cycloplegic medication until the condition stabilized. As the double AC did not resolve, we opted for observation instead of rebubbling. During follow-up, we still observed progressive corneal edema with multiple epithelial bullae and persistent double AC.

**Fig. 3:**
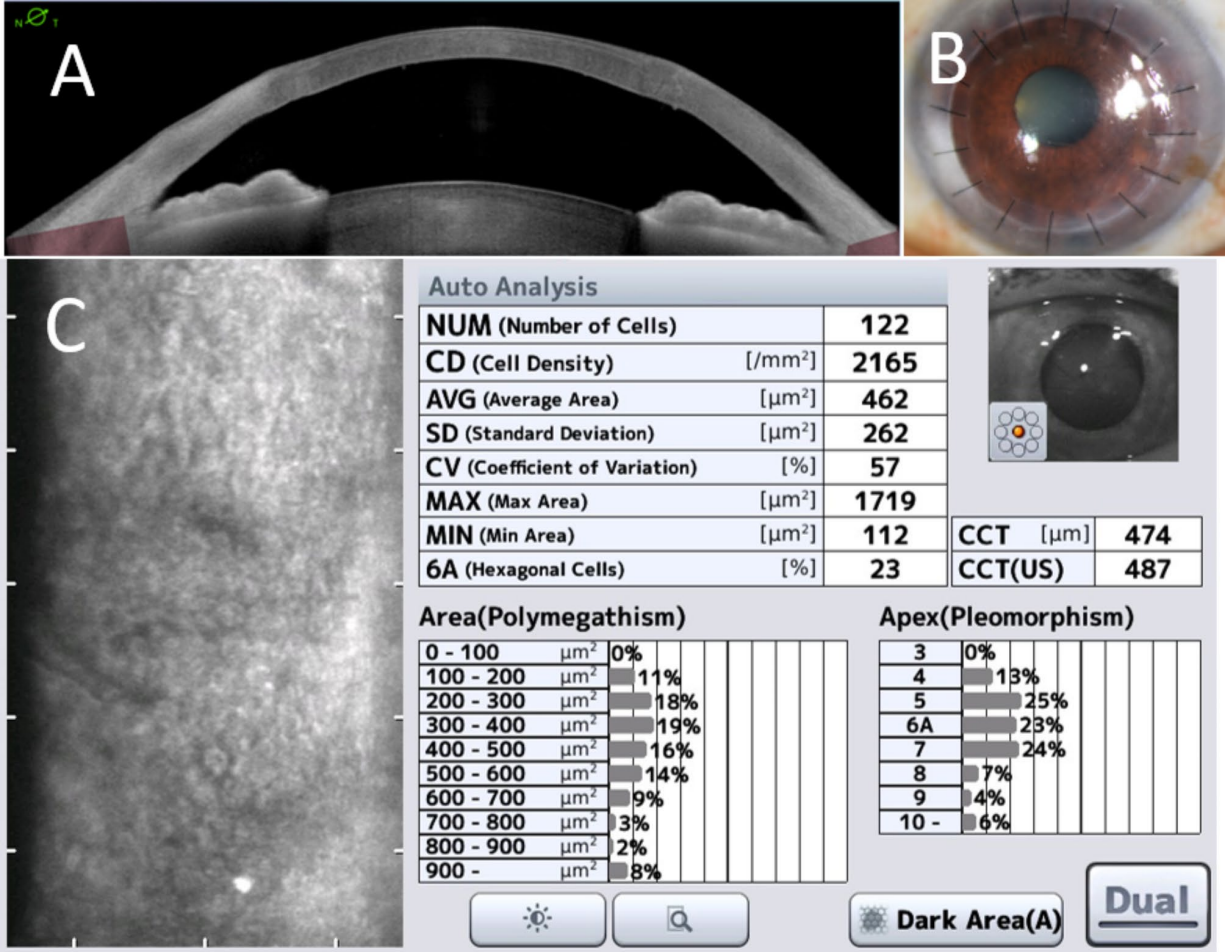
Anterior segment optical coherence tomography (**A**), the anterior segment photo (**B**), and specular microscopy (**C**) confirmed the complete resolution of Descemet membrane-endothelium complex detachment and corneal edema

At two months post-operation, the double AC space gradually became shallower and completely closed at three months, resulting in a transparent cornea. AS-OCT and the anterior segment photo demonstrated the complete resolution of the DMD, as shown in Fig. 3A and B. Pachymetry measured 474 microns, and endothelial cell density was 2,165 cells/ mm^2^ (Fig. 3C).

## Discussion and conclusions

DMD is a potential vision-threatening complication that can occur after various surgeries, for example, cataract surgery, trabeculectomy, vitrectomy, and corneal transplantation [8]. A double AC was the most common early postoperative complication after DALK [5]. This complication typically arises from DM perforation [5], although it can also occur without visible perforation. In such cases, potential causes include residual viscoelastic material in the interface, a type 2 bubble, the donor-recipient curvature mismatch, or retained endothelium in the donor graft.

In our case, DMD was observed on postoperative day 1 without any intraoperative DM tear, as confirmed by reviewing surgical video and analyzing AS-OCT images. We concluded that an unrecognized type 2 BB was the cause of postoperative double AC. We did not use viscoelastic material and ensured complete removal of the DM from the donor graft, thereby excluding other common causes of double AC as previously reported. The possible mechanism is that, in the presence of a type 2 BB, air penetration into the anterior chamber created a temporary false pathway through the trabecular meshwork, allowing aqueous humor to flow back through the pathway. Another contributing mechanism could be temporary endothelial dysfunction caused by intraoperative manipulation and intermittent rises in intraocular pressure during bubble re-injection. Additionally, one theory explaining the pathogenesis of non-rhegmatogenous DMD after type 2 BB creation suggests that mechanical stretching of the junctional endothelial intercellular complexes may compromise the integrity and barrier properties of the endothelium, impairing its regulation of water and electrolytes [9]. Lastly, given the complex nature of the surgery, including failed big bubble attempts and conversion to manual dissection, microperforations during manual dissection could have occurred and should be considered as potential contributing factors to the development of DMD.

Currently, evidence-based guidelines for managing double AC without DM perforation following DALK are not well established. As a result, management strategies are largely guided by rational concepts, literature reviews, and personal experience [10]. Surgeons typically decide between observation, descemetopexy (with or without interface fluid drainage), or re-grafting procedures, with the latter being more invasiveness. Observation allows for spontaneous DM attachment and is non-invasive, but delayed intervention may lead to DM fibrosis, reducing the chance of successful descemetopexy [11]. Descemetopexy, while aiming to prevent the need for a more invasive second keratoplasty, may result in the loss of over 20% of endothelial cells [7]. An unsuccessful tamponade could further lower endothelial cell counts, potentially delaying or diminishing the chances of spontaneous resolution. In severe cases, repeated gas tamponades might reduce endothelial cell density, leading to corneal decompensation and unavoidable re-grafting.

Optimal observation duration and criteria for proceeding with more invasive procedures are yet to be clearly defined. In our case, due to extensive DM detachment, we initially attempted descemetopexy using air with an inferior PI, expecting reattachment after a single rebubbling. However, the development of intraoperative malignant glaucoma prevented successful descemetopexy. We proceeded with observation and medication. Despite persistent double AC, progressive corneal edema, and multiple epithelial bullae, we adhered to the “wait and see” technique. DM gradually reattached, and corneal edema resolved spontaneously within three months.

According to the literature review, there have been seven cases of non-rhegmatogenous DMD following DALK [9, 12–16]. Three cases were initially managed with a “wait and observe” approach, resulting in spontaneous resolution at 1 and 3 weeks, although the exact time of resolution was not reported for one case [9, 12]. Another three cases proceeded with early air [13, 14] or gas [15] tamponade with [13] or without interface fluid drainage in conjunction with graft repositioning [14, 15]. However, all three cases were failed by postoperative day 1. These cases were managed conservatively without further intervention, and DM eventually reattached over time. Another case of DMD was complicated by acute angle-closure glaucoma caused by a large type 2 bubble remaining in the pre-Descemet membrane layer, requiring urgent surgical intervention [16]. 

Theories suggest that the second AC resolves after the spontaneous closure of the false pathway through the trabecular meshwork and restoration of endothelial function. Therefore, attempting to hasten adherence by pushing up the dysfunctional DM-endothelial complex through descemetopexy may be prone to failure [9, 15], unlike rhegmatogenous DMD, where sealing the perforation is more straightforward. A limitation of the current evidence is that it is based only on case reports.

In the case of recognized type 2 BB, the recommendation is to completely remove the stroma using the proper technique without evacuating the first bubble. If a significantly large bubble persists, the peripheral pre-Descemet’s membrane layer should be punctured to collapse the bubble [17]. The bubble test can be used to confirm the presence of a BB and ensure complete stromal removal [18, 19]. Surgical video recordings and AS-OCT were valuable tools for identifying possible causes and classifying complications. They also assist in the appropriate management, monitoring, and prevention of postoperative DMD complications.

In conclusion, early postoperative double AC without visible DM perforation after DALK, and unexplained by residual viscoelastic material, curvature mismatch, or retained donor endothelium, may resolve spontaneously. Therefore, the “wait and observe” approach is a safe and reasonable primary management strategy, and it can also be considered as a secondary option after descemetopexy failure before proceeding to more invasive keratoplasty. As there is no established timing for intervention, further studies are needed to determine the management guidelines.

## Data Availability

All data and images can be obtained by contacting the corresponding author.

## Abbreviations

AC: Anterior chambers
DMD: Descemet membrane detachment
DALK: Deep anterior lamellar keratoplasty
DM: Descemet membrane
PKP: Penetrating keratoplasty
BB: Big-bubble
RE: Right eye
BCVA: Best-corrected visual acuity
LE: Left eye
PI: Peripheral iridotomy
